# The effects of COVID-19 vaccines on economic activity

**DOI:** 10.1186/s41937-021-00082-0

**Published:** 2022-01-12

**Authors:** Pragyan Deb, Davide Furceri, Daniel Jimenez, Siddharth Kothari, Jonathan D. Ostry, Nour Tawk

**Affiliations:** 1 International Monetary Fund, Washington, DC 20431 USA; 2 Centre for Economic Policy Research, London, UK

**Keywords:** COVID-19, Pandemics, Vaccinations, Containment measures, C31, C33, E65, O50, F4

## Abstract

This paper empirically examines the economic effects of COVID-19 vaccine rollouts using a cross-country daily database of vaccinations and high-frequency indicators of economic activity—nitrogen dioxide (NO_2_) emissions, carbon monoxide (CO) emissions, and Google mobility indices—for a sample of 46 countries over the period December 16, 2020 to June 20, 2021. Using surprises in vaccines administered, we find that an unexpected increase in vaccination per capita is associated with a significant increase in economic activity. We also find evidence for nonlinear effects of vaccines, with the marginal economic benefits being larger when vaccination rates are higher. Country-specific conditions play an important role, with lower economic gains if strict containment measures are in place or if the country is experiencing a severe outbreak. Finally, the results provide evidence of spillovers across borders, highlighting the importance of equitable access to vaccines across nations.

## Introduction

Since the beginning of the COVID-19 pandemic, countries have been forced to put in place stringent non-pharmaceutical interventions (henceforth referred to as containment measures) in order to limit the spread of the virus. But these containment measures have come at enormous economic costs, resulting in unprecedented economic losses (Carvalho et al. 2020; Coibion et al., 2020; Deb et al., 2020b; IMF, 2020a, 2020b), despite wide-scale fiscal measures launched worldwide to mitigate some of these losses (Deb et al., 2021d). With the advent of vaccines, the focus of countries has shifted towards vaccinating their populations against the Coronavirus (SARS-nCOV-2) as quickly as possible, in an effort to raise immunity against the virus and ease containment measures, thereby helping their economies recover.

Evidence from the epidemiological literature has already established the effectiveness of COVID-19 vaccines in reducing virus transmission, curbing severe infections and hospitalizations, and lowering fatalities (Dagan et al., 2021; Polack et al., 2020, Voysey et al. 2021). However, with wide access to vaccines having only picked up since early 2021, there is thus far little empirical evidence on the effects of vaccine rollouts on economic activity in a cross-country setting (see for example, Deb et al., 2021b for an analysis based on regional data for a more limited set of countries). In this context, this paper complements our analysis of the health impact of vaccinations (Deb et al., 2021a), by providing an empirical assessment of the effects of COVID-19 vaccinations on high-frequency proxies of economic activity for a sample of 46 countries over the period December 16, 2020 to June 20, 2021. It then goes on to study potential nonlinearity in the economic effect of vaccines, including depending on the initial rate of vaccination and other country-specific conditions, such as the stringency of containment measures or the severity of the COVID-19 outbreak. Finally, the paper examines how vaccinations and new COVID-19 cases in a country’s main trading partners can affect its local economic activity through economic inter-linkages.

For this purpose, we assemble a daily database on high-frequency indicators of economic activity—nitrogen dioxide (NO_2_) emissions, carbon monoxide (CO) emissions, and Google mobility indices. We use NO_2_ emissions as our baseline measure for economic activity as it is most highly correlated with lower frequency measures of economic activity like industrial production and PMI indices. Economic activity data is combined with daily data on COVID-19 vaccines administered per capita (one or two doses), confirmed COVID-19 infections and containment measures. We rely on high-frequency identification to establish causality, controlling for lagged effects of economic and health variables, and accounting for expectations about the country-specific evolution of the pandemic using a set of controls that can affect future infections and economic activity—non-pharmaceutical interventions (NPIs) such as containment measures, enhanced testing, contact tracing, and public information campaigns aimed at increasing social awareness—and country-specific time trends. To further identify the causal link between COVID-19 vaccines and economic activity, we construct a novel measure of surprises in vaccines administered. The variable is computed by taking the difference between the share of population that is fully vaccinated and the predicted share that is expected to be fully vaccinated (see Data section) based on production and procurement of vaccines.

We find that surprises in vaccinations have significant economic effects: a 10% of population (unexpected) increase in vaccine is associated with an increase in daily per capita NO_2_ emissions of about 0.3 standard deviation (an increase of almost 30% relative to its median). To better put this result in perspective, this is broadly equivalent to going from a full lockdown (stringency index of 1) to containment measures equivalent to a stringency level of 0.5. This comparison, however, likely underestimates the economic gains from higher vaccination rates as we find evidence for nonlinear effects of vaccines, with the marginal economic benefits being larger when vaccination rates are higher. Similar positive effects are obtained for the impact of vaccinations on mobility indicators and CO (which is only significant with a lag).

In addition, we find that country-specific conditions play an important role in determining the economic impact of vaccines. Economic gains are lower if strict containment measures are in place, as they constrain economic activity even with vaccinations picking up. Similarly, economic gains are lower if the country is experiencing a severe outbreak during the vaccine rollout as people continue to voluntarily socially distance till cases come down significantly.

Finally, we find evidence of spillovers across borders: an increase in COVID-19 cases in trading partner countries results in a slowdown in domestic economic activity due to spillovers from trade. Furthermore, an increase in COVID-19 vaccinations in the main trading partner countries has a positive and statistically significant effect on domestic economic activity. These results highlight the importance of equitable and speedy access to vaccines across nations, as higher vaccination rates in trading partners not only improve health outcomes in partners (Deb et al., 2021a), but are also likely to improve economic conditions domestically.

Our paper contributes to two main strands in the literature. The first is that which studies the economic effects of COVID-19 vaccines and remains somewhat limited. Sandmann et al. (2021) examine the potential health and economic value of COVID-19 vaccinations in the UK and find that introducing vaccinations leads to a reduction in community transmission and incremental monetary gains from a health-care perspective. Deb et al. (2021b) employ a regional database of 17 countries (326 states) to study the impact of COVID-19 vaccinations on economic activity proxies—night-time lights, aerosol optical depth (AOD) emissions, and mobility. They find that vaccine deployment has persistent positive effects on the level of economic activity. Agarwal and Gopinath (2021) propose a cost–benefit analysis for an expedited rollout of vaccines in an equitable manner across all countries, and find that while vaccinating 40 percent of the world’s population by 2021 could cost around $50 billion, its engendered benefits could reach about $9 trillion in economic gains. This paper contributes to this literature by: (1) extending on Deb et al. (2021b) to examine the effects of surprises in vaccines administered on economic activity for 43 countries; (2) studying the role of country-specific conditions in amplifying/dampening the effects of vaccine surprises; and (3) examining the impact of COVID-19 cases and vaccines in main trading partners on a country’s own economic activity levels.

This paper also contributes to the literature which uses high-frequency indicators to proxy economic activity. Fernández-Villaverde and Jones (2020) and Sampi et al. (2020) establish that Google mobility data is an adequate proxy for economic activity by finding a high correlation between GDP and mobility data. Deb et al., (2020a, 2020b) use Google mobility indicators to capture the economic impact of the COVID-19 pandemic. Lin and McElroy (2011) show that variation in NO_2_ emissions in China resembles its GDP growth during and after the Global Financial Crisis. Deb et al. (2020b) quantify the economic costs of the COVID-19 pandemic using nitrogen dioxide (NO_2_) emissions and estimate losses in NO_2_ emissions 30 days after the implementation of containment measures to be equivalent to about a 15% loss in industrial production. This paper contributes to this strand of the literature by putting together a novel database of daily high-frequency indicators of NO_2_ emissions, CO emissions, and Google mobility indicators for 43 countries, to examine how they are affected by surprises in vaccines administered.

The paper is structured as follows. Section 2 describes the data and Sect. 3 the methodological approach. Section 4 discusses the results on effects of vaccinations on economic outcomes, the role of country-specific factors, and the effects of COVID-19 cases and vaccines in main trading partners on a country’s own economic activity. The last section concludes.

## Data

Our empirical analysis relies on a comprehensive country-level database of daily COVID-19 cases and vaccinations, high-frequency proxies of economic activity (NO_2_, CO, and mobility), and government responses to the pandemic in the form of different non-pharmaceutical interventions. Appendix Table 7 provides a summary of the data used.

### COVID-19 related variables

*COVID-19 vaccines* data is sourced from the Our World in Data COVID-19 repository.^1^ Vaccines data is disaggregated by first and second shots, with data covering up to 202 countries starting in December 2020.

*COVID-19 cases:* Daily data on COVID-19 cases is collected from the COVID-19 Data Repository by the Center for Systems Science and Engineering (CSSE) at Johns Hopkins University.^2^ Coverage begins from January 22, 2020 for 208 countries.

*Expected vaccinations:* Data on expected vaccination rollout is taken from Airfinity, a science information and analytics company.^3^ Airfinity uses a supply-driven model to construct country-level daily time series for the number of people expected to be fully vaccinated. Their model tracks vaccine production facilities and links supply from each facility to actual and expected deliveries to each country, including through international sharing arrangement like the COVAX facility. Based on actual/expected deliveries, Airfinity produces a time series for people expected to be fully vaccinated, allowing for different speeds of vaccine rollout depending on the income level of the country. In addition, while their model predicts the expected number of people fully vaccinated, it takes into account country specific policies, such as greater emphasis on first doses in some countries like Canada, Finland, or the UK.

*Vaccine surprise:* We construct a novel measure of vaccine surprises by taking the difference between actual vaccination rates (percent of population fully vaccinated) in the data and expected vaccination rates. The vaccine surprise variable has two key advantages over simply using vaccination rates in our empirical analysis. Economic activity is more likely to increases following surprises in vaccination rates rather than to actual vaccination rates in the population (after controlling for number of cases) as economic agents will likely internalize expected vaccine rollouts. In other words, if future vaccine rollouts are anticipated, using actual vaccination rates would lead to underestimating the economic effect of vaccines (for a similar argument, related to fiscal policy actions see Ramey, 2011). In addition, surprises in vaccination are less likely to be endogenous to economic developments and COVID-19 trends, as well as other shocks affecting vaccine supply, allowing for better causal identification.

To check that indeed our vaccine surprise variable can be deemed as exogenous, we analyze the relationship between the vaccine surprise and other variables affecting high-frequency indicators of economic activity.^4^ The results reported in Appendix Table 8 show that the vaccine surprise variable is uncorrelated with daily contemporaneous developments related to the pandemic (new cases and stringency of containment measures), the procurement of vaccines, as well as lags of our high-frequency measures of economic activity (NO_2_, CO, mobility discussed below).

**Fig. 1:**
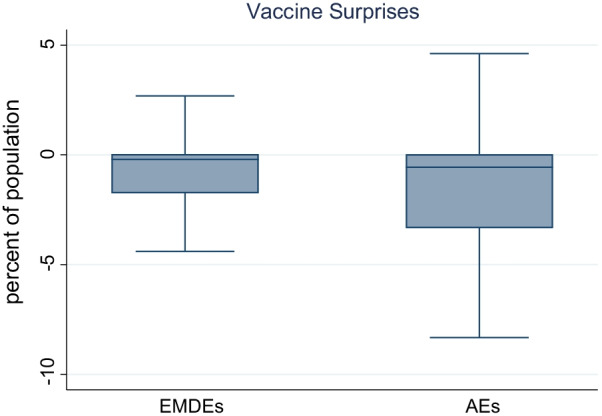
Vaccine surprises, people fully vaccinated (percent of population). Source: Airfinity, Our World in Data, IMF staff calculations

**Fig. 2:**
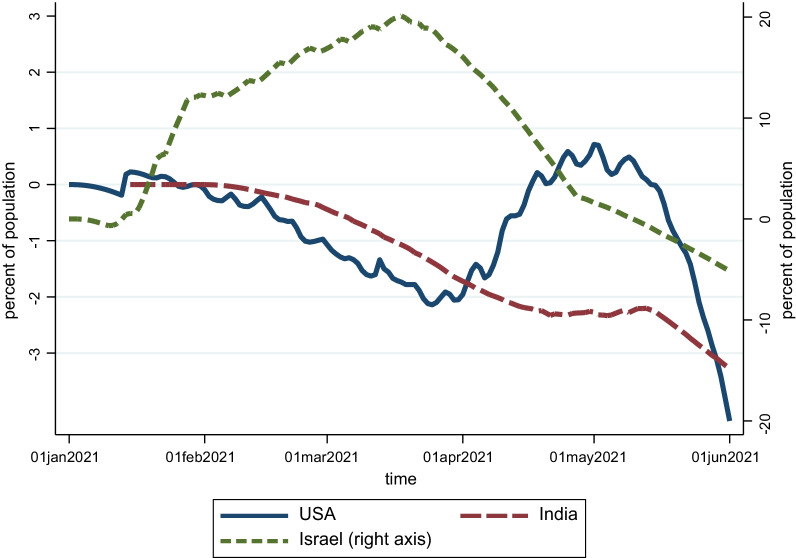
Vaccine surprises, people fully vaccinated (percent of population). Source: Airfinity, Our World in Data, IMF staff calculations

Finally, we looked at the time series of vaccine surprises as well as their distribution across countries. Figure 1 shows the box plot for the vaccine surprise variable for advanced economies as well as emerging and developing economies. The median vaccine surprise is close to zero for both groups, although the distribution is skewed with larger negative surprises indicating, consistent with anecdotal evidence, slower than expected vaccine rollout. Figure 2 shows the time series for the vaccine surprise variable for a few specific countries. The USA has had relatively small surprises, with vaccinations lagging model predictions till April, but a subsequent pickup in rollout resulting in actual vaccination rates catching up to model predictions. By contrast, vaccine rollout has consistently underperformed model predictions in India and overperformed model predictions in Israel.

### Economic activity variables

*Emissions Data:* Following Deb et al. (2020b), we use emissions as a proxy for economic activity. We gather nitrogen dioxide (NO_2_) and carbon monoxide (CO) emissions data at a daily frequency from The World Air Quality Index project, a non-profit project whose mission is to provide a unified worldwide air quality information.^5^ Our sample covers 64 countries for NO_2_ and 59 countries for CO starting on January 1st, 2021. The data is reported using EPA standards and is based on the median emissions reported by city-specific stations which are updated three times a day.

*Mobility:* We collect retail and recreation mobility data from Google Mobility Community Reports, which have been shown to be a good proxy for economic activity (IMF, 2020a).^6^ The reports provide country-level daily data by country and highlight the percent change in visits to places related to retail and recreation activity (e.g. restaurants, cafes, shopping centers, movie theaters, museums, and libraries). The data is reported as the change relative to a pre-pandemic baseline value for that corresponding day of the week, said baseline is calculated as the median value for that corresponding day of the week, during the five-week period between January 3rd and February 6th, 2020. Daily data is available for 135 countries in our dataset, with coverage beginning from February 15th, 2020.

While the daily indicators used for the analysis do not capture every aspect of economic activity, they are strongly correlated with more traditional, monthly measures of economic activity such as industrial production (IP), Purchasing Managers Index (PMI), and OECDs composite leading indicator (CLI). Table 1 shows a statistically significant relationship using a monthly database of indicators from January 2019 to June 2021. We find that an increase in all 3 indicators is associated with a corresponding pickup in the average level of NO_2_ emissions during the estimation period. The results for CO emissions and mobility also go in the same direction but are statistically weaker, suggesting that NO_2_ is a more robust proxy of economic activity.

**Table 1:** High-frequency indicators and monthly measures of economic activity (2019–2021)

Variables	(1)	(2)	(3)	(4)	(5)	(6)	(7)	(8)	(9)
	NO_2_	NO_2_	NO_2_	CO	CO	CO	Mobility	Mobility	Mobility
Industrial production (IP)	0.365665*** (0.077)			0.173088*** (0.059)			8.497571* (4.251)		
Purchasing Managers' Index (PMI)		0.253454*** (0.085)			− 0.082399 (0.085)			2.700382 (3.941)	
Composite leading indicator (CLI)			0.004974** (0.002)			0.003803 (0.002)			1.040853*** (0.365)
Constant	− 0.011543 (0.035)	0.011421 (0.037)	− 0.463284** (0.189)	− 0.016109 (0.043)	0.001686 (0.038)	− 0.398601 (0.237)	0.258797 (1.670)	− 0.913749 (1.740)	− 102.413798*** (36.859)
Observations	1178	1070	1017	976	956	803	944	918	592
R-squared	0.262	0.201	0.213	0.119	0.061	0.074	0.574	0.519	0.623
Country FE	Yes	Yes	Yes	Yes	Yes	Yes	Yes	Yes	Yes
Time FE	Yes	Yes	Yes	Yes	Yes	Yes	Yes	Yes	Yes
No. of countries	43	39	37	38	37	31	57	54	37

Standard error clustered at the country level. Robust standard errors in parentheses ***, **, and * represent statistically significant at 1, 5, and 10%, respectively

We further establish that NO_2_ emissions are also strongly associated with the level of economic activity over a longer horizon. Using data available from the OECD database for total man-made emissions of nitrogen oxides from 1990 to 2018, we test the sensitivity of such emissions to conventional measures of economic activity such as GDP growth, growth in manufacturing value added and growth in measures of industrial production. Table 2 shows a robust relationship between these economic variables and NO_2_ emissions.

**Table 2:** NO_2_ emissions and economic activity—historical data (1990–2018)

	(1)	(2)	(3)	(4)	(5)	(6)	(7)	(8)	(9)
GDP growth	0.341**	0.326*	0.307*						
	(2.147)	(1.942)	(1.865)						
Manufacturing VA growth				0.130***	0.134***	0.135***			
				(3.347)	(3.426)	(3.334)			
IP growth							0.203*	0.201**	0.206**
							(2.028)	(2.166)	(2.381)
Time trend	− 0.001***	− 0.001	0.000	− 0.002***	− 0.001**	− 0.001	− 0.002**	0.000	0.001
	(− 3.353)	(− 1.520)	(0.770)	(− 3.352)	(− 2.086)	(− 1.046)	(− 2.348)	(0.638)	(0.919)
Average temperature		− 0.012***	− 0.011**		− 0.011***	− 0.011**		− 0.010**	− 0.012**
		(− 3.285)	(− 2.521)		(− 3.151)	(− 2.628)		(− 2.214)	(− 2.537)
Urban population		− 0.004			− 0.004			− 0.011**	
		(− 1.335)			(− 1.324)			(− 2.088)	
Population density			− 0.001*			− 0.001*			− 0.002**
			(− 1.920)			(− 1.896)			(− 2.097)
Income per-capita			0.000			0.000			− 0.000
			(0.065)			(0.108)			(− 1.200)
Log GDP			− 0.056						
			(− 1.601)						
Log manufacturing VA						0.005			
						(0.295)			
Log IP									− 0.042
									(− 1.356)
Constant	− 0.005	0.350*	1.763*	0.004	0.380*	0.101	0.006	0.913**	0.558**
	(− 0.529)	(1.898)	(1.825)	(0.500)	(1.838)	(0.195)	(0.399)	(2.509)	(2.511)
Fixed effects	Yes	Yes	Yes	Yes	Yes	Yes	Yes	Yes	Yes
Clustered SE	Yes	Yes	Yes	Yes	Yes	Yes	Yes	Yes	Yes
R-squared	0.061	0.082	0.086	0.051	0.074	0.076	0.058	0.100	0.092
Observations	929	863	828	852	789	775	623	568	566
No. of countries	36	36	36	36	36	36	30	30	30

Standard error clustered at the country level. Robust standard errors in parentheses ***, **, and * represent statistically significant at 1, 5, and 10%, respectively

Summarizing, the results in Tables 1 and 2 validate our choice of NO_2_ emissions as the main proxy of interest for the empirical work in this paper.

### Government responses

*Containment measures:* We use data from Oxford’s COVID-19 Government Response Tracker (OxCGRT).^7^ OxCGRT collects information on government policy responses across eight dimensions, namely: (1) school closures; (2) workplace closures; (3) public event cancellations; (4) gathering restrictions; (5) public transportation closures; (6) stay-at-home orders; (7) restrictions on internal movement; and (8) international travel bans. The database scores the stringency of each measure ordinally, for example, depending on whether the measure is a recommendation or a requirement and whether it is targeted or nation-wide. We normalize each measure to range between 0 and 1 to make them comparable. In addition, we compute and aggregate a Stringency Index as the average of the sub-indices, again normalized to range between 0 and 1. The data starts on January 1, 2020 and covers 151 countries/regions.

## Methodology

We conduct three distinct exercises to study: (1) the impact of vaccines on economic outcomes; (2) the heterogeneity in the impact of vaccines depending on country conditions; and (3) the effects from increased COVID-19 infections in main trading partners on economic activity.

### Effect of vaccinations on economic outcomes

For the analysis of the economic impact of vaccinations, we use our country-time panel dataset at the daily frequency that allows for high-frequency identification of the impact of vaccinations on economic outcomes. Establishing causality is difficult because vaccine rollout may depend on current or expected economic conditions, either directly or through the evolution of the pandemic which in turn impacts economic activity. We try to mitigate reverse causality by controlling for lagged values of number of COVID-19 cases as well as lagged values of our high-frequency economic indicators. We also control for country fixed effects which effectively control for vaccine procurement, structural factors (such as health capacity) affecting the speed of vaccine rollout, and for differences of the structure economic activity across countries—such as the share services or tourism. To further account for expectations about country-specific evolution of vaccine rollout and economic activity, we also control for a set of variables which may affect future infections and economic activity such as non-pharmaceutical interventions (NPIs)—including containment measures—and country-specific time trends.^8^ We include time fixed effects to account for global factors affecting the evolution of the virus (such as new variants), vaccination (supply disruptions), and economic activity (global shifts in confidence).

As a first step, we use an econometric specification as follows:

1 $$\Delta Y_{i,t} = \mu_{i} + \gamma_{t} + \beta V_{i,t - l} + \theta X_{i,t - l} + \varepsilon_{i,t}$$

where ${Y}_{i,t}$ alternatively denotes: the level of NO_2_ emissions as a share of the population, the level of CO emissions as a share of the population, and Google’s retail mobility indicator, of country *i* at time *t*. ${V}_{i,t-l}$ denotes the share of the individuals in the population which have been vaccinated. The coefficient $\beta$ gives us the impact of higher vaccination rates on various economic variables. We include country and time fixed effects (${\mu }_{i}$ and ${\gamma }_{t}$) to control for country-specific characteristics and global trends that can affect the evolution of the pandemic. We also include a vector of control variables,${X}_{i,t-l}$, which comprises of the lagged level of COVID-19 cases, the lagged levels of NO_2_ emissions, CO emissions, mobility, and stringency of containment measures, as well as country-specific time trends. We opt for a one-day lags as a baseline to reduce the risk of reverse causality but examine various lags as a robustness check.

We also explore nonlinear effects of vaccines by adding the square of ${V}_{i,t-l}$ in some specifications.

Despite the extensive set of controls used in Eq. (1), residual concerns about endogeneity may remain. To further address this issue, we construct a novel measure of vaccine surprises that accounts for expected rollout given procurement and use this as the independent variable instead of actual vaccination rates. We test the impact of unexpected vaccinations by modifying the econometric specification as follows:

2 $$\Delta Y_{i,t} = \mu_{i} + \gamma_{t} + \beta SV_{i,t - l} + \theta X_{i,t - l} + \varepsilon_{i,t}$$

where ${SV}_{i,t-l}$ is a measure of vaccine surprises constructed by taking the difference between the share of people that are fully vaccinated and the expected share as predicted by Airfinity’s supply-driven vaccine rollout model (see Sect. 2 for details). All other variables are the same as in Eq. (1).

### Role of country-specific conditions on the effect of vaccines on economic activity

We also test the role of country-specific conditions in shaping the effects of vaccinations on economic activity. Namely, we examine whether the impact of vaccines on economic outcomes varies depending on the stringency of containment measures, or the severity of the outbreak itself. For this, we use a semi-parametric approach in which we interact vaccination surprises with quartiles (“bins”) of country-specific conditions. This approach does not impose the strong parametric restriction of the effectiveness of vaccines changing linearly with country conditions. Rather, it allows us to flexibly explore variation in vaccine effectiveness across the distribution of country conditions.^9^ We augment Eq. (2) with the following:

3 $$\begin{aligned} & \Delta Y_{i,t} \,=\, \mu_{i} + \gamma_{t} + \beta_{1} Q_{1} *SV_{i,t - l} + \beta_{2} Q_{2} *SV_{i,t - l} + \beta_{3} Q_{3} *SV_{i,t - l} + \beta_{4} Q_{4} *SV_{i,t - l} \\ & \quad + \mathop \sum \limits_{j = 1}^{4} \delta_{j} Q_{j} + \theta X_{i,t - l} + \varepsilon_{i,t} \\ \end{aligned}$$

where ${Q}_{1}$, ${Q}_{2}$, ${Q}_{3},$ and ${Q}_{4}$ are dummy variables that denote alternatively quartiles of the stringency of containment measures, or the level of new COVID-19 cases in a country. Quartiles are interacted with our vaccine surprises variable. Interaction terms are also lagged 1 day, consistent with the vaccine surprise variable. If the coefficients on the interaction terms of higher quartiles differ from those at lower quartiles, it signifies that the effectiveness of vaccines depends on country-specific conditions. In the robustness checks, we also test for alternative nonlinear specifications such as those based on linear interactions and smooth transition functions.

### Effect of COVID-19 cases and vaccines in trading partners on economic outcomes

We further test whether a pandemic outbreak in a countries’ close trading partners can affect economic activity locally. Similarly, we also explore whether increased vaccines administered in a country’s main trading partners can help boost economic activity locally. To investigate whether this may be the case, we create the following:

4 $$Trading\, Partner_{i,t} = \mathop \sum \limits_{j = 1}^{N} w_{i,j} *Outcomes_{j,t}$$

where ${Trading\, Partner}_{i,t}$ is a term which alternatively denotes the COVID-19 cases or COVID-19 vaccinations in country *i’s* main trading partners. ${Outcomes}_{j,t}$ refer to either country *j’*s COVID-19 cases or vaccinations as a share of population at time *t.* These outcomes are combined with ${w}_{i,j},$ trade weights constructed based on bilateral trade flows (exports and imports from the 2019 Directional of Trade Statistics) between country *i* and country *j* that are scaled by total exports and imports such that $\sum_{j=1}^{n-i}{w}_{i,j}$=1. The weights thus capture each country’s relative trade exposure to its different trading partners, and the spillover term ${Trading Partner}_{i,t}$ captures COVID-19 cases/vaccines in a country’s trading partners, assigning higher weights to countries with strong trade linkages under the assumption that countries with closer trading relationships will have a larger impact on domestic economic activity.^10^ This term is introduced to Eq. (2) as following:

5 $$\Delta Y_{i,t} =\, \alpha + \mu_{i} + \gamma_{t} + \beta SV_{i,t - l} + \gamma Trading\, Partner_{j,t - m} + \theta X_{i,t - l} + \varepsilon_{i,t}$$

Equations (1) through (5) are estimated using OLS, with standard errors clustered at the country level.

## Results

### Baseline results

We begin by assessing the impact of vaccinations on high-frequency proxies of economic activity—the level of nitrogen dioxide (NO_2_) emissions, the level of carbon monoxide (CO) emissions, and the decline in retail and recreation mobility. Table 3 column 1 shows estimates for Eq. (1) with change in NO_2_ as the dependent variable. The coefficient on vaccinations is positive and significant, indicating that higher vaccination rates are associated with an increase in economic activity. Next, we introduce the second vaccine dose as an additional variable and find that second vaccine doses also have a significant effect on NO_2_ emissions (Table 3, column 2).

**Table 3:** Effect of vaccines on economic activity—alternative high-frequency economic indicators

Variables	(1)	(2)	(3)	(4)	(5)	(6)	(7)	(8)	(9)	(10)	(11)	(12)
	NO_2_	NO_2_	NO_2_	NO_2_	CO	CO	CO	CO	Mobility	Mobility	Mobility	Mobility
First dose per capita	0.004539** (0.002)	0.002492* (0.001)		0.004790*** (0.002)	0.000123 (0.001)	− 0.000001 (0.001)		0.000273 (0.001)	0.070539 (0.114)	− 0.003033 (0.089)		0.058095 (0.110)
Second dose per capita		0.010261*** (0.003)				0.000626 (0.001)				0.379216** (0.144)		
Surprises in vaccines administered (per capita)			0.011232*** (0.002)	0.011086*** (0.002)			0.000538 (0.000)	0.000530 (0.000)			0.496498** (0.217)	0.494948** (0.216)
COVID-19 cases per capita (lag)	0.005300 (0.025)	− 0.017239 (0.021)	0.003334 (0.019)	− 0.023925 (0.023)	0.001749 (0.008)	0.000369 (0.008)	0.001265 (0.007)	− 0.000291 (0.008)	0.009952 (1.358)	− 0.832376 (1.163)	− 1.315359 (1.069)	− 1.647660 (1.445)
NO_2_ emissions per capita (lag)	− 0.554868*** (0.031)	− 0.560258*** (0.030)	− 0.565083*** (0.033)	− 0.566872*** (0.033)	0.008407 (0.010)	0.008073 (0.010)	0.008540 (0.010)	0.008438 (0.010)	0.342440 (0.488)	0.154355 (0.416)	0.188923 (0.435)	0.167182 (0.427)
CO emissions per capita (lag)	0.019537 (0.044)	0.022281 (0.048)	0.018653 (0.046)	0.018753 (0.045)	− 0.516948*** (0.115)	− 0.516781*** (0.115)	− 0.508693*** (0.121)	− 0.508687*** (0.120)	− 0.086799 (0.992)	0.006510 (1.079)	− 0.139745 (0.986)	− 0.137757 (0.979)
Containment measures (lag)	− 0.228008 (0.144)	− 0.161003 (0.125)	− 0.197816 (0.119)	− 0.159762 (0.121)	− 0.065146 (0.044)	− 0.061008 (0.042)	− 0.068454 (0.046)	− 0.066262 (0.044)	− 50.95811*** (9.110)	− 48.48066*** (8.067)	− 49.0482(7.160) 9***	− 48.57127*** (7.039)
Mobility (lag)	0.001094 (0.001)	0.000755 (0.001)	0.000741 (0.001)	0.000713 (0.001)	− 0.000268 (0.000)	− 0.000289 (0.000)	− 0.000325 (0.000)	− 0.000327 (0.000)	− 0.595574*** (0.059)	− 0.608155*** (0.057)	− 0.619935*** (0.058)	− 0.620257*** (0.058)
Constant	− 9.944917 (11.214)	− 38.42561*** (10.513)	6.795432 (5.203)	− 6.379479 (6.577)	2.156221 (2.085)	0.419049 (1.855)	2.113492 (1.511)	1.363800 (2.304)	− 1151.16493* (602.222)	− 2212.384*** (798.360)	− 765.8508*** (273.347)	− 925.315016* (459.661)
Observations	6226	6226	5879	5879	6208	6208	5861	5861	6215	6215	5868	5868
R-squared	0.326	0.330	0.332	0.333	0.281	0.281	0.278	0.278	0.432	0.437	0.443	0.443
Country FE	Yes	Yes	Yes	Yes	Yes	Yes	Yes	Yes	Yes	Yes	Yes	Yes
Time FE	Yes	Yes	Yes	Yes	Yes	Yes	Yes	Yes	Yes	Yes	Yes	Yes
Health controls and country-time trends	Yes	Yes	Yes	Yes	Yes	Yes	Yes	Yes	Yes	Yes	Yes	Yes
No. of countries	46	46	44	44	46	46	44	44	46	46	44	44

Table reports results for Eq. (1). The dependent variable is NO_2_ emissions per capita for columns 1–4, CO emissions per capital for columns 5–8 and change in retail and recreation mobility for columns 9–12. The regressions control for stringency of containment measures, other non-pharmaceutical interventions and health policy controls (one lag), lags of mobility (one lag), lagged new cases, (one lag), lagged NO_2_ and CO emissions (one lag) country-specific time trends, as well as country and time fixed effects. Standard errors are clustered at the country level. ***, **, and * represent statistically significant at 1, 5, and 10%, respectively

The impact of vaccination on economic activity based on Eq. (1) might, however, be biased due to residual concerns regarding endogeneity. As discussed before, to address this, we focus on surprises in vaccines administered (per capita) measured as the difference between actual vaccinations and the expected vaccination rollout, which we show is more likely to be exogenous. We find that surprises in vaccinations are strongly associated with higher NO_2_ emissions (Table 3, column 3).^11^ The magnitude of the coefficient of vaccine surprise is typically larger than those of first and second doses suggesting that expectations play a role. Quantitatively, a surprise increase in vaccinations by 10% of population is associated with an increase in daily per capita NO_2_ emissions of around 0.112, which is about a 0.28 standard deviation increase in NO_2_, or an increase of almost 30 percent relative to median.^12^ As expected, stronger containment measures are also associated with lower economic activity. Taking the results at face value they imply that a 10-percentage points vaccine surprise has about the same impact on economic activity as going from a full lockdown (stringency index of 1) to containment measures equivalent to a stringency level of 0.5.^13^

Columns 4 through 8 of Table 3 repeat the analysis with changes in CO as the dependent variable while columns 9 through 12 use mobility as the dependent variables. The results for CO are statistically weaker and generally not significant at one lag. The insignificant impact on CO at short horizons may reflect the weaker correlation between CO and other measures of economic activity documented in Table 1. As discussed below, the impact on CO becomes significant at longer lags. For mobility measures, vaccine surprises have a significant positive impact, with a 10-percentage points vaccine surprise associated with an increase in mobility of 5 points (Table 3, column 11), which is equivalent to the average difference in mobility in the USA in March 2021 when a bulk of containment measures remained in place versus mobility in May 2021 when access to vaccines improved and states started easing restrictions gradually.

### Lag structure

**Fig. 3:**
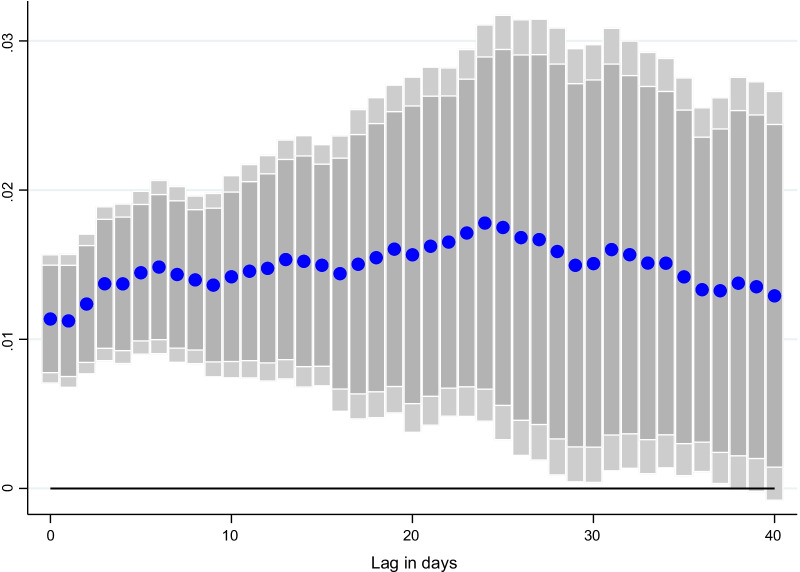
Effect of vaccinations on NO_2_ emissions per capita, at different lags. Coefficient $\beta$ is reported for each lag ℓ (1–40), and based on $\Delta {Y}_{i,t}=\alpha + {\mu }_{i}+{\gamma }_{t}+{\beta SV}_{i,t-l}+ {\theta X}_{i,t-l}+ {\varepsilon }_{i,t}$ for a sample of 44 countries using daily data from December 20, 2020–June 16, 2021. ${Y}_{i,t}$ denotes NO_2_ emissions per capita and ${SV}_{i,t-l}$ is the surprises in vaccines administered (per capita).${\mu }_{i}$ and ${\gamma }_{t}$ are the country and time fixed effects. *X* is a vector of control variables which includes the level of new cases, NO_2_ and CO emissions per capita, the stringency of containment measures index, and mobility indices at *t-1*. Lightly shaded bars denote 90 percent confidence bands, and dark-shared bars denote 95 percent confidence bands

**Fig. 4:**
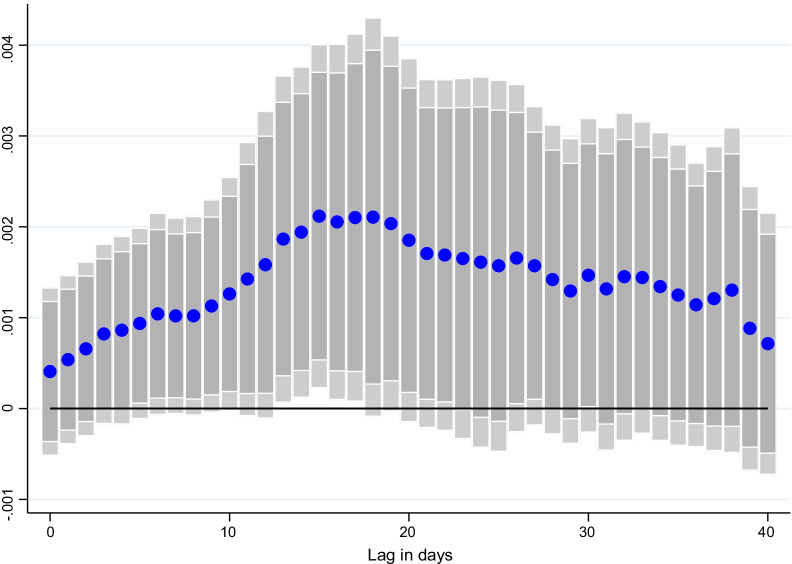
Effect of vaccinations on CO emissions per capita, at different lags. Coefficient $\beta$ is reported for each lag ℓ (1–40), and based on $\Delta {Y}_{i,t}=\alpha + {\mu }_{i}+{\gamma }_{t}+{\beta SV}_{i,t-l}+ {\theta X}_{i,t-l}+ {\varepsilon }_{i,t}$ for a sample of 44 countries using daily data from December 20, 2020–June 16, 2021. ${Y}_{i,t}$ denotes CO emissions per capita and ${SV}_{i,t-l}$ is the surprises in vaccines administered (per capita).${\mu }_{i}$ and ${\gamma }_{t}$ are the country and time fixed effects. *X* is a vector of control variables which includes the level of new cases, NO_2_ and CO emissions per capita, the stringency of containment measures index, and mobility indices at *t-1*. Lightly shaded bars denote 90 percent confidence bands, and dark-shared bars denote 95 percent confidence bands

**Fig. 5:**
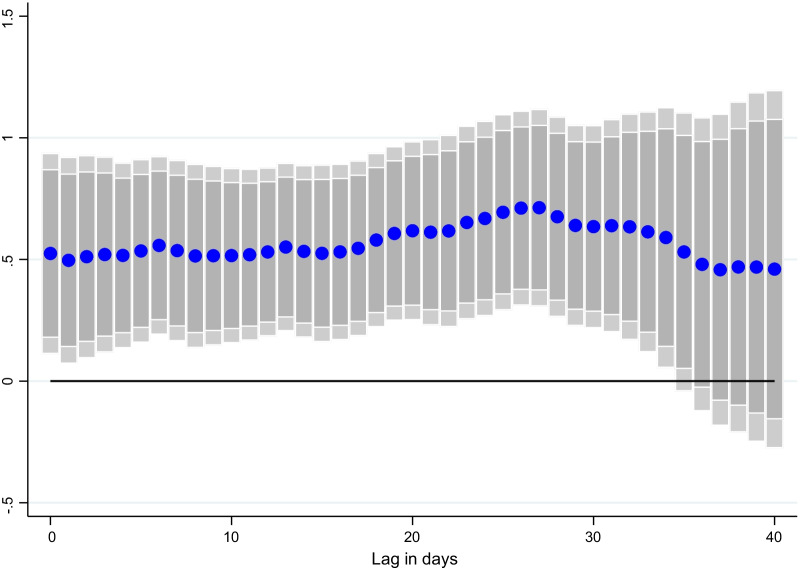
Effect of vaccinations on mobility, at different lags. Coefficient $\beta$ is reported for each lag ℓ (1–40), and based on $\Delta {Y}_{i,t}=\alpha + {\mu }_{i}+{\gamma }_{t}+{\beta SV}_{i,t-l}+ {\theta X}_{i,t-l}+ {\varepsilon }_{i,t}$ for a sample of 44 countries using daily data from December 20, 2020–June 16, 2021. ${Y}_{i,t}$ denotes changes in retail and recreation mobility and $S{V}_{i,t-l}$ is the surprises in vaccines administered (per capita).${\mu }_{i}$ and ${\gamma }_{t}$ are the country and time fixed effects. *X* is a vector of control variables which includes the level of new cases, NO_2_ and CO emissions per capita, the stringency of containment measures index, and mobility indices at *t-1*. Lightly shaded bars denote 90 percent confidence bands, and dark-shared bars denote 95 percent confidence bands

Turning to the lag structure, Figs. 3, 4, and 5 show that the impact of vaccination increases with greater lags, as economic agents gain greater protection from the virus and increasingly resume economic activities. In particular, the results for CO, which are not statically significant immediately, become significant over time.

### Other robustness

The results are robust to different subsamples. Appendix Table 10 summarizes the robustness results for NO_2_: (1) the results hold when the data is winsorized to ensure that the results are not driven by outliers; (2) our results go through if we drop countries that started vaccinating late such as Colombia and Vietnam—started their vaccination campaigns after March 1; (3) the results are also robust to dropping countries that started vaccinations very early such as USA and UK—already reached 5% of the population by February 1; and (4) the results hold if we drop one region at a time or one country at a time, suggesting that they are not driven by a particular region or country with high levels of vaccinations (such as the USA, the UK, or Israel).

### Nonlinear effects

Next, we test for nonlinearities in the impact of vaccines on economic activity. Economic activity may not respond significantly to vaccinations in the initial phase of the vaccine rollout which often targets specific groups (health-care workers or the elderly). As the rollout continues and vaccinations spread to a wider set of people, confidence is more likely to improve, in part because individuals are more willing to reduce voluntary social distancing as the risk of infections go down.

Column 1 of Table 4 reports results for change in NO_2_ as the dependent variable, where we include the share of population that has received one vaccine dose as well as the square of this variable as independent variables. We find evidence for nonlinear effects, with the square term being positive and significant, indicating that the economic benefits of vaccinations are larger when vaccination rates increase. Similar nonlinear effects are seen for the second vaccine dose (Table 4, column 3).

**Table 4:** Nonlinear effects of vaccines on economic activity

Variables	(1)	(2)	(3)	(4)	(5)	(6)
	NO_2_	NO_2_	NO_2_	NO_2_	NO_2_	NO_2_
First dose per capita	− 0.002795	− 0.000146	0.004753***			
	(0.003)	(0.003)	(0.001)			
First dose squared	0.000131*	0.000052				
	(0.000)	(0.000)				
Second dose per capita		0.008875***	0.000145	0.010812*		
		(0.003)	(0.003)	(0.006)		
Second dose squared			0.000214***			
			(0.000)			
Surprises in vaccines administered (per capita)				− 0.008556	− 0.008666	
				(0.009)	(0.010)	
Second dose per capita * Surprises in vaccines administered				0.000289**		
				(0.000)		
*Interaction with second dose quartiles (1st quartile omitted)*						
2nd Quartile of second doses administered * Surprises in vaccines					0.024510**	
					(0.011)	
3rd Quartile of second doses administered * Surprises in vaccines					0.025691**	
					(0.011)	
4th Quartile of second doses administered * Surprises in vaccines					0.019766*	
					(0.010)	
Low second doses administered * Surprises in vaccines						− 0.009003
						(0.010)
High second doses administered * Surprises in vaccines						0.016742***
						(0.004)
Observations	6226	6226	6226	5879	5879	5879
R-squared	0.328	0.330	0.331	0.335	0.335	0.333
Country FE	Yes	Yes	Yes	Yes	Yes	Yes
Time FE	Yes	Yes	Yes	Yes	Yes	Yes
No. of countries	46	46	46	44	44	44
*P*-value F-test						0.0578

Table reports results for the nonlinear impact of vaccines on NO_2._ Columns 1 through 3 add additional terms for the square of the share of population that is vaccinated to the specification in Eq. (1). Column 4 through 6 test use different specification to test for interactions between the share of population fully vaccinated and the vaccine surprise variable. Column 4 uses a simple interaction term, column 5 is based on different quartiles of the vaccination rate (Eq. 2), while column 6 allows for interactions based on a smooth transition function. The regressions control for stringency of containment measures, other non-pharmaceutical interventions and health policy controls (one lag), lags of mobility (one lag), lagged new cases, (one lag), lagged NO2 and CO emissions (one lag) country-specific time trends, as well as country and time fixed effects. Standard errors are clustered at the country level. ***, **, and * represent statistically significant at 1, 5, and 10%, respectively

Columns 4–6 of Table 4 test for nonlinear effects of our more exogenous vaccine surprise variable and finds similar results. Column 4 allows for the simple interaction between the vaccine surprise variable and the share of population that is fully vaccinated. The interaction term is positive and significant, indicating that vaccine surprises have larger economic effects when the level of vaccinations is higher. In column 5, we present the results obtained by interacting the vaccine surprise variable with different quartiles of the share of people fully vaccinated, while in column 6 those by interacting vaccination rates with the surprise variable using a smooth transition function. Results are similar across specifications.

Appendix Tables 11 and 12 repeat the nonlinear regressions with changes in CO and mobility as the dependent variable, respectively. Results for these other indicators are generally less precisely estimated compared to NO_2_.^14^

### Role of containment measures and severity of outbreak

This section examines the extent to which the impact of vaccines on economic outcomes depends on other factors such as the stringency of containment measures and the severity of the outbreak.

### Stringency of containment measures

In addition to the impact of vaccines, economic activity is also dependent on the severity of non-pharmaceutical interventions in the form of containment measures. A quick vaccine rollout may not lead to an immediate improvement in economic outcomes if strong containment measures need to be maintained at the same time. Column 1 of Table 5 adds an interaction term between the vaccine surprise term and the stringency of containment measures categorized into quartiles (Eq. 3). The interaction terms are negative for the higher quartiles and significantly different from zero for the 4th quartile. This indicates that an increase in vaccines leads to a smaller positive impact on NO_2_ emissions when accompanied with stringent containment measures, potentially because movement restrictions prevent individuals from ramping up economic activity in response to higher vaccination rates. Column 3 of Table 5 repeats the analysis using retail mobility as the dependent variable. The results are similar to the NO_2_ regressions.

**Table 5:** Effect of vaccines on economic activity—role of containment new cases

Variables	(1)	(2)
	NO_2_	NO_2_
Surprises in vaccines administered (per capita)	0.014502*** (0.003)	0.036355** (0.014)
*Interaction with stringency measures quartiles (1st quartile omitted)*		
2nd Quartile of Containment Measures * Surprises in vaccines	− 0.001964	
	(0.001)	
3rd Quartile of Containment Measures * Surprises in vaccines	− 0.005628	
	(0.004)	
4th Quartile of Containment Measures * Surprises in vaccines	− 0.012473**	
	(0.005)	
*Interaction with new cases quartiles (1st quartile omitted)*		
2nd Quartile of New Cases * Surprises in vaccines		− 0.020599
		(0.012)
3rd Quartile of New Cases * Surprises in vaccines		− 0.029437**
		(0.014)
4th Quartile of New Cases * Surprises in vaccines		− 0.026988*
		(0.014)
Observations	5859	5859
R-squared	0.334	0.335
Country FE	Yes	Yes
Time FE	Yes	Yes
Country-Time Trend	Yes	Yes
No. of countries	44	44

Table reports results for Eq. 3. The dependent variable is change in NO_2_ per capita in columns 1 and 2 and change in retail and recreational mobility in columns 3 and 4. The forecast error in vaccine rollout is interacted with the stringency of containment measures (categorized into four quartiles) in column 1 and 3. The forecast error in vaccine rollout is interacted with the level of new cases (moving average over seven days and also categorized into four quartiles) in column 2 and 4. The variable are lagged one day. All regressions control for stringency of containment measures and other non-pharmaceutical interventions, lagged mobility and NO_2_ per capita, country specific time trends, as well as country and time fixed effects. Standard errors are clustered at the country level. ***p < 0.01, **p < 0.05, *p < 0.1

### Severity of the outbreak

The impact of vaccines on economic activity is also likely to depend on the stage of the outbreak. If a country is in the middle of a large outbreak, an increase in vaccine rollout may have only a muted impact on activity as people continue to voluntarily socially distance till cases come down significantly. To test this hypothesis, column 2 of Table 5 adds an interaction term between the vaccine surprise variable and the number of new cases (moving average over seven days) in the country categorized into quartiles (Eq. 2). The interaction terms are negative and significant for the higher quartiles, supporting the hypothesis that voluntary social distancing may limit the beneficial impact of vaccines on economic activity when cases are high. Results are qualitatively similar when using mobility as the dependent variable, though statistically weaker.^15^

### Spillovers from foreign COVID-19 cases and vaccines to economic activity

The pace of COVID-19 vaccinations across countries has been uneven, producing divergent economic and health outcomes across nations. In this section, we explore whether global health and vaccination outcomes can have an indirect effect on a country’s own economic activity levels through economic linkages such as trade. We also investigate whether the rapid vaccination pace in a systemically important economy such as the USA would have any spillover effects to the rest of the world.

### Effect of foreign COVID-19 cases on local economic activity

**Fig. 6:**
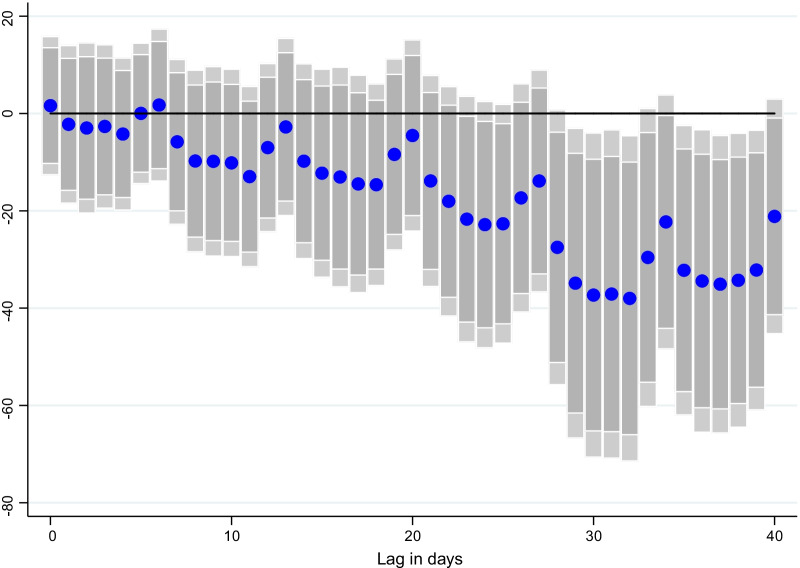
Effect of foreign new COVID-19 cases on economic activity. Coefficient $\gamma$ is reported for each lag ℓ (1–40), and based on $\Delta {Y}_{i,t}=\alpha + {\mu }_{i}+{\gamma }_{t}+{\beta SV}_{i,t-l}+\gamma {Trading Partner}_{j,t-m}+ {\theta X}_{i,t-l}+ {\varepsilon }_{i,t}$ for a sample of 43 countries using daily data from December 20, 2020–June 16, 2021. where ${Y}_{i,t}$ denotes: the number of new COVID-19 cases. ${Trading Partner}_{i,t}$ is a spillover term for COVID-19 cases in main trading partner countries. ${SV}_{i,t-l}$ denotes the share of the individuals in the population which have received at least one vaccine shot.${\mu }_{i}$ and ${\gamma }_{t}$ are the country and time fixed effects. *X* is a vector of control variables which includes the level of new cases, NO_2_ and Co emissions per capita, the stringency of containment measures index, and mobility indices at *t-1*. ℓ denotes the lags in the response of new COVID-19 cases. Lightly shaded bars denote 90 percent confidence bands, and dark-shared bars denote 95 percent confidence bands

Deb et al. (2021a) find that neighboring COVID-19 cases can have a significant effect on a country’s own pandemic, amplifying its own caseload despite vaccinations or containment measures. Similarly, we find significant spillover effects on country *i*’s economic activity, proxied by NO_2_ emissions, through an increase in new COVID-19 cases in its main trading partners (Table 6, column 1). Namely, a one standard deviation increase in foreign COVID-19 cases would lead to a 0.10 standard deviation decrease in domestic NO_2_ emissions, effectively reducing domestic economic activity through traditional economic linkages. This effect is lagged (Fig. 6), with the impact becoming statistically significant after around 21 days. This is plausible, given that the negative effects of an outbreak on a country’s economic activity are likely to take time to materialize, which implies that negative spillover effects would show after some lag.

**Table 6:** Effects of foreign new COVID-19 cases and vaccines on economic activity

	(1)	(2)	(3)
	NO_2_ (per capita)	NO_2_ (per capita)	NO_2_ (per capita)
Surprises in vaccines administered (per capita)	0.009579***	0.010250***	0.011306***
	(0.002)	(0.003)	(0.002)
Foreign new COVID-19 cases per capita (trade weighted)	− 37.319212**		
	(17.053)		
Foreign vaccines administered per capita (trade weighted)			0.068932**
			(0.028)
US Vaccinations per capita (trade weighted)		0.247082*	
		(0.126)	
COVID-19 cases per capita (lag)	0.026458	0.030142	0.014836
	(0.016)	(0.025)	(0.020)
NO_2_ emissions per capita (lag)	− 0.568909***	− 0.550169***	− 0.566578***
	(0.033)	(0.026)	(0.033)
CO emissions per capita (lag)	0.013661	− 0.010112	0.022937
	(0.044)	(0.030)	(0.049)
Containment measures (lag)	− 0.180132	− 0.329187**	− 0.224757*
	(0.141)	(0.136)	(0.131)
Mobility (lag)	0.000825	0.000120	0.000774
	(0.001)	(0.001)	(0.001)
	(0.020)	(0.021)	(0.022)
Constant	3.056202	9.155523	− 2.330700
	(5.401)	(10.212)	(6.549)
Observations	5807	4606	5807
R-squared	0.334	0.334	0.333
Country FE	Yes	Yes	Yes
Time FE	Yes	Yes	Yes
Health controls and country-time trends	Yes	Yes	Yes
No. of countries	43	42	43

Table reports results for Eq. (4). The dependent variable is NO_2_ emissions per capita. A spillover term (foreign COVID-19 cases/foreign vaccinations/US vaccinations) (lag 30 days) is introduced to the equation to alternately capture the effects of trading partners’ COVID-19 new cases or vaccines on a country’s economic activity using bilateral trade weights (Eq. 3). The regressions control for stringency of containment measures, other non-pharmaceutical interventions and health policy controls (one lag), lags of mobility (one lag), lagged new cases, (one lag), lagged NO_2_ and CO emissions (one lag) country-specific time trends, as well as country and time fixed effects. Standard errors are clustered at the country level. ***, **, and * represent statistically significant at 1, 5, and 10%, respectively

### Effect of foreign COVID-19 vaccines on local economic activity

**Fig. 7:**
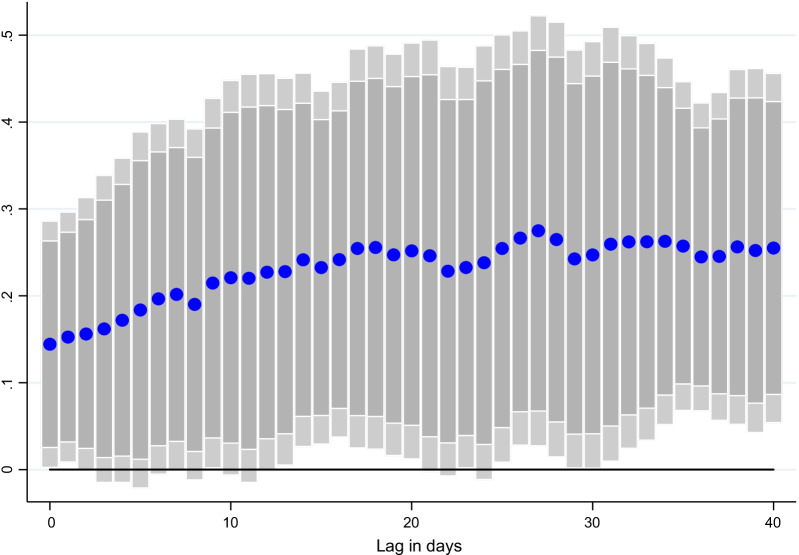
Effect of US COVID-19 vaccinations on economic activity. Coefficient $\gamma$ is reported for each lag ℓ (1–40), and based on $\Delta {Y}_{i,t}=\alpha + {\mu }_{i}+{\gamma }_{t}+{\beta SV}_{i,t-l}+\gamma {Trading Partner}_{j,t-m}+ {\theta X}_{i,t-l}+ {\varepsilon }_{i,t}$ for a sample of 43 countries using daily data from December 20, 2020–June 16, 2021. where ${Y}_{i,t}$ denotes: the number of new COVID-19 cases. ${Trading Partner}_{i,t}$ is a spillover term for COVID-19 vaccinations in the USA. ${SV}_{i,t-l}$ denotes the share of the individuals in the population which have received at least one vaccine shot. ${\mu }_{i}$ and ${\gamma }_{t}$ are the country and time fixed effects. *X* is a vector of control variables which includes the level of new cases, NO_2_ and Co emissions per capita, the stringency of containment measures index, and mobility indices at *t-1*. ℓ denotes the lags in the response of new COVID-19 cases. Lightly shaded bars denote 90 percent confidence bands, and dark-shared bars denote 95 percent confidence bands

While foreign COVID-19 cases can have a dampening effect on local economic activity, the opposite seemingly holds for foreign COVID-19 vaccinations. First, we look at the spillovers from US vaccinations, given the relatively high rate of vaccinations in the USA and the important global economic linkages. Table 6, column 2 adds a US spillover term to the regression which is calculated by multiplying US vaccination rates with each country’s bilateral trade exposure to the USA. We find that there are positive spillover effects from increased vaccinations in the USA.^16^ Figure 7 shows the effect of US vaccinations on NO_2_ emissions at different lags, with economic gains materializing with a 20-day lag.

**Fig. 8:**
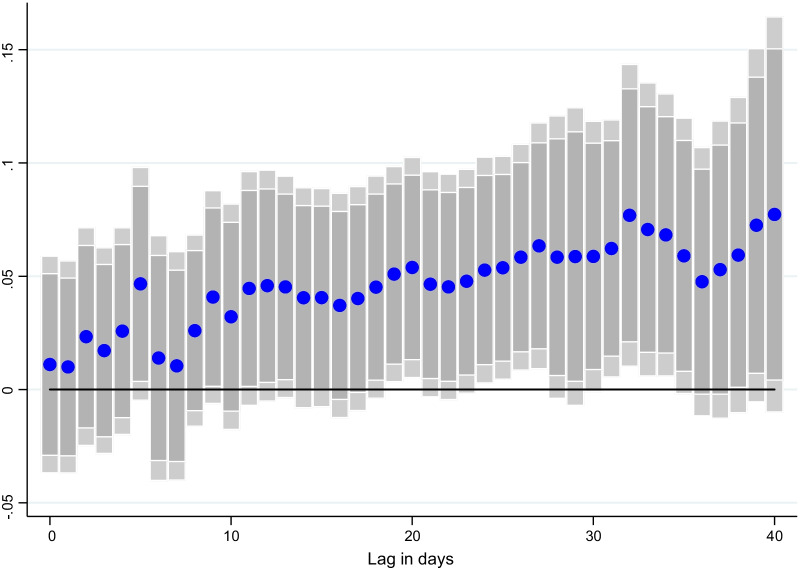
Effect of foreign COVID-19 vaccines on economic activity. Coefficient $\gamma$ is reported for each lag ℓ (1–40), and based on $\Delta {Y}_{i,t}=\alpha + {\mu }_{i}+{\gamma }_{t}+{\beta SV}_{i,t-l}+\gamma {Trading Partner}_{j,t-m}+ {\theta X}_{i,t-l}+ {\varepsilon }_{i,t}$ for a sample of 43 countries using daily data from December 20, 2020–June 16, 2021. where ${Y}_{i,t}$ denotes: the number of new COVID-19 cases. ${Trading Partner}_{i,t}$ is a spillover term for COVID-19 vaccinations in main trading partner countries. $S{V}_{i,t-l}$ denotes the share of the individuals in the population which have received at least one vaccine shot. ${\mu }_{i}$ and ${\gamma }_{t}$ are the country and time fixed effects. *X* is a vector of control variables which includes the level of new cases, NO_2_ and Co emissions per capita, the stringency of containment measures index, and mobility indices at *t-1*. ℓ denotes the lags in the response of new COVID-19 cases. Lightly shaded bars denote 90 percent confidence bands, and dark-shared bars denote 95 percent confidence bands

Next, we look at spillovers from vaccinations more broadly by adding the average vaccination rate of trading partners as described in Eq. 4 and 5. Results in Table 6 column 3 show that foreign COVID-19 vaccines have a positive and statistically significant effect on economic activity, with one standard deviation increase in foreign COVID-19 vaccines leading to a 0.13 standard deviation increase in NO_2_ emissions. The result is persistent, and also materializes with a lag: Fig. 8 shows the impact of trading partner vaccination at different lags, with a persistently higher trend again materializing around the 20-day mark. The results provide additional evidence that a higher vaccination pace worldwide can also boost domestic economic activity.

Agarwal and Gopinath (2021) stress the importance of vaccinating a large share of the global population as quickly as possible, noting that “the pandemic is not over anywhere unless it is over everywhere.” The economic spillover results in this paper and the health spillover results in Deb et al. (2021a), Deb et al. (2021b), Deb, Ganslmeier, et al. (2021)) provide strong empirical evidence in support of such a policy, showing that a virus outbreak in trading partners is likely to have severe negative health and economic consequences domestically. Thus, ensuring an even distribution of vaccines, especially by sharing any vaccine oversupply in advanced economies, can help bring the pandemic to a speedier end, minimizing the global loss of lives and promoting a robust economic recovery.

## Conclusion

Vaccines against the coronavirus disease are key to exiting the health and economic crises that COVID-19 has brought about. In this paper, we provide an empirical assessment of the effects of COVID-19 vaccines on economic activity. We put together a novel daily database of high-frequency economic indicators—NO_2_ emissions, CO emissions, and Google mobility indices, combined with data on new COVID-19 cases, vaccinations, and surprises in vaccines administered, as well as data on non-pharmaceutical intervention. To the best of our knowledge, this is the first empirical assessment of the economic effects of COVID-19 vaccine surprises on a large-scale sample (46 countries), and of the role of country-specific conditions and the impact of COVID-19 vaccines and cases in main trading partners on a country’s local economic activity.

The results suggest that COVID-19 vaccines have a large and statistically significant effect on economic activity. A surprise increase in vaccinations by 10% of population is associated with an increase in daily per capita NO_2_ emissions of about 0.3 standard deviations (an increase of almost 30 percent relative to its median). This is equivalent to going from a full lockdown (stringency index of 1) to containment measures equivalent to a stringency level of 0.5. We get similar significant results for mobility, with a 10-percentage points vaccine surprise associated with an increase in mobility of 5 percentage points. CO emissions also increase in response to increased vaccine surprises, but with a lag. These results are robust to alternative specifications and, as our result show, the magnitude of the effect is likely to increase with higher vacation rates.

We also find that the effect of COVID-19 vaccines on economic activity varies depending on the level of stringency measures imposed in a country, as well as on the severity of the pandemic outbreak in a country. Namely, the results suggest that the impact of vaccines rollouts may not lead to an immediate improvement in economic outcomes if strong containment measures need to be maintained at the same time. In addition, we find that the effect of vaccines on economic activity is more muted when a country is experiencing a severe outbreak, or when COVID-19 cases are high.

Finally, the results provide evidence on the importance of an even access to vaccines across nations, as we find that countries can be affected by pandemic spillovers via trade linkages with their main trading partners. Namely, we find that while COVID-19 cases in main trading partner countries can dampen local economic activity in a country, rollouts of vaccines have the opposing effect, boosting local economic activity and thus speeding up the global recovery. This highlights the potential gains from vaccine sharing, and the importance of vaccinating early and broadly not only a country’s own population, but all populations, in order to bring a swifter end to the global economic crisis the pandemic had brought about.^17^

The findings in this paper, combined with results from Deb et al., (2021a, 2021b) on the beneficial effects of vaccines on health outcomes, highlight the importance of vaccines to address the crisis instigated by the COVID-19 pandemic (see also IMF, 2021). In addition to the direct health and economic benefits of vaccines, this paper finds evidence for the dampening effect of containment measures and local outbreaks on economic activity, and the importance of sharing excess vaccine doses to boost global economic activity. We hope the empirical analysis provides evidence to policymakers on the importance of vaccinating swiftly and efficiently, both locally and globally, in order to exit the COVID-19 crisis.

## Data Availability

The authors would make them available after acceptance.

## Appendix

See Tables 7 , 8 , 9 , 10, 11, 12, and 13.

**Table 7:** Summary statistics

	Obs	Mean	Std. Dev	Min	Max	Source	Starting date	N. of countries
*Panel A**: **Summary statistics for daily time-varying data*								
First dose per 100 inhabitants	23,257	13.26	17.73	0.00	116.15	OWID	16-Dec-20	202
Second dose per 100 inhabitants	15,257	9.59	14.27	0.00	114.86	OWID	27-Dec-20	180
Surprises in vaccines administered	18,845	− 0.62	5.70	− 24.26	54.50	Airfinity	27-Dec-20	182
COVID 19 cases per 100 inhabitants	71,969	0.01	0.02	0.00	1.83	JHU	23-Jan-20	205
NO2 per 1 M inhabitants	31,529	1.37	3.42	0.00	66.84	AqiCN	1-Jan-20	62
CO per 1 M inhabitants	27,828	0.54	1.51	0.00	25.73	AqiCN	1-Jan-20	57
Mobility	63,740	− 21.66	25.07	− 100.00	181.00	Google	15-Feb-20	135

	Obs	Mean	Std. Dev	Min	Max	Source	Date	N. of countries
*Panel B**: **Summary statistics for monthly time-varying data*								
IP	1115	3,058.62	21,972.43	10.94	238,183.40	Haver Analytics	Jan-20	63
PMI	990	50.29	7.58	20.68	72.80	Haver Analytics	Jan-20	55
CLI	663	97.86	3.46	64.75	103.59	OECD	Jan-20	39
NO_2_	419	1.31	2.55	0.01	21.00	AqiCN	Dec-20	61
CO	383	0.48	1.23	0.00	10.19	AqiCN	Dec-20	57
Mobility	2271	− 20.15	23.16	− 90.33	176.33	Google	1-Feb	135

**Table 8:** Bivariate regressions of vaccine surprises with various variables

Variables	(1)	(2)	(3)	(4)	(5)	(6)	(7)	(8)	(9)	(10)	(11)	(12)
New cases	14.6677											
	(16.078)											
Containment measures		− 4.7802										
		(5.014)										
Confirmed vaccine procurement (cumulative)			42.6610									
			(83.119)									
Potential vaccine procurement (cumulative)				25.5848								
				(80.705)								
Confirmed vaccine procurement (new)					− 64.1929							
					(39.836)							
Potential vaccine procurement (new)						− *55.4293*						
						(39.495)						
NO_2_ (lag 1)							0.0199					
							(0.040)					
NO_2_ (lag 30)								0.0693				
								(0.068)				
CO (lag 1)									− 0.0431			
									(0.048)			
CO (lag 30)										− 0.0293		
										(0.031)		
Mobility (lag 1)											0.0000	
											(0.001)	
Mobility (lag 30)												0.0036
												(0.003)
Observations	5875	5848	5805	5805	5805	5805	5866	5790	5847	5700	5875	5875
R-squared	0.442	0.445	0.459	0.451	0.447	0.447	0.436	0.465	0.436	0.467	0.438	0.438
Country FE	Yes	Yes	Yes	Yes	Yes	Yes	Yes	Yes	Yes	Yes	Yes	Yes
Time FE	Yes	Yes	Yes	Yes	Yes	Yes	Yes	Yes	Yes	Yes	Yes	Yes
No. of countries	44	44	44	44	44	44	44	43	44	44	44	44

Table reports results from regressing the vaccine surprise variable (difference between percent of population that is fully vaccinated and the expected share that will be fully vaccinated taken from Airfinity) on various variables. All regressions include country and time fixed effects. Standard errors are clustered at the country level. ***, **, and * represent statistically significant at 1, 5, and 10%, respectively

**Table 9:** Robustness checks—instrumenting using vaccine surprises

Variables	(1)	(2)	(3)
	NO_2_	CO	Mobility
Second dose per capita (instrumented with vaccine surprise)	0.01141***	0.00062	0.51819*
	(0.002)	(0.000)	(0.263)
COVID-19 cases per capita (lag)	− 0.00497	0.00239	− 1.72696
	(0.019)	(0.007)	(1.232)
NO_2_ emissions per capita (lag)	− 0.56732***	0.00825	0.09591
	(0.032)	(0.010)	(0.379)
CO emissions per capita (lag)	0.01742	− 0.50816***	− 0.22019
	(0.048)	(0.121)	(1.062)
Containment measures (lag)	0.00070	− 0.00033	− 0.61985***
	(0.001)	(0.000)	(0.058)
Mobility (lag)	− 0.19556	− 0.07154	− 48.75752***
Observations	5909	5891	5898
R-squared	0.287	0.258	0.305
Country FE	Yes	Yes	Yes
Time FE	Yes	Yes	Yes
Health controls and country-time trends	Yes	Yes	Yes
No. of countries	44	44	44

Table reports results for Eq. (1) but instruments for the share of fully vaccinated individuals with the vaccine surprise variable. The dependent variable is NO_2_ emissions per capita for column 1, CO emissions per capital for column 2, and change in retail and recreation mobility for column 3. The regressions control for stringency of containment measures, other non-pharmaceutical interventions and health policy controls (1 lag), lags of mobility (1 lag), lagged new cases, (1 lag), lagged NO2 and CO emissions (1 lag) country-specific time trends, as well as country and time fixed effects. Standard errors are clustered at the country level. ***, **, and * represent statistical significant at 1, 5, and 10%, respectively

**Table 10:** Robustness checks—baseline using NO_2_ emissions

Variables	(1)	(2)	(3)	(4)	(5)	(6)	(7)	(8)
		Drop late	Drop early	Without	Without	Without	Without	Without
	1% winsorize	start after March 1	5% before February 1	APD	EUR	MCD	WHD	AFR
Surprises in vaccines administered (per capita)	0.011918***	0.011192***	0.008219***	0.010750***	0.008216**	0.010365***	0.012232***	0.011289***
	(0.003)	(0.002)	(0.003)	(0.003)	(0.003)	(0.002)	(0.003)	(0.002)
COVID-19 cases per capita (lag)	0.004288	0.003674	− 0.019220	0.004979	0.020262	0.007671	− 0.007044	0.002204
	(0.019)	(0.020)	(0.025)	(0.019)	(0.019)	(0.019)	(0.021)	(0.020)
NO_2_ emissions per capita (lag)	− 0.564944***	− 0.565331***	− 0.582338***	− 0.560856***	− 0.606884***	− 0.548998***	− 0.567048***	− 0.565005***
	(0.033)	(0.033)	(0.035)	(0.037)	(0.035)	(0.034)	(0.033)	(0.033)
CO emissions per capita (lag)	0.018059	0.018016	0.008404	0.020323	0.041983	0.016742	0.008033	0.017675
	(0.046)	(0.046)	(0.050)	(0.046)	(0.062)	(0.100)	(0.048)	(0.046)
Containment measures (lag)	− 0.207061	− 0.229113*	− 0.211049	− 0.151022	− 0.354375**	− 0.130982	− 0.252608	− 0.203953
	(0.123)	(0.129)	(0.138)	(0.129)	(0.155)	(0.128)	(0.155)	(0.122)
Mobility (lag)	0.000842	0.000757	0.000698	0.000383	0.000399	0.001335**	0.000582	0.000699
	(0.001)	(0.001)	(0.001)	(0.001)	(0.001)	(0.001)	(0.001)	(0.001)
Constant	5.976847	6.610367	− 62.995520***	8.405028	− 5.091505	11.078580**	− 5.697044	7.415563
	(5.387)	(5.448)	(6.169)	(5.249)	(7.801)	(5.231)	(8.123)	(5.213)
Observations	5879	5624	5158	4878	2629	5422	4823	5759
R-squared	0.332	0.333	0.353	0.341	0.344	0.340	0.343	0.333
Country FE	Yes	Yes	Yes	Yes	Yes	Yes	Yes	Yes
Time FE	Yes	Yes	Yes	Yes	Yes	Yes	Yes	Yes
Health controls and country-time trends	Yes	Yes	Yes	Yes	Yes	Yes	Yes	Yes
No. of countries	44	41	39	34	23	40	36	43

Table reports results for Eq. (1). The dependent variable is NO_2_ emissions per capita. The regressions control for stringency of containment measures, other non-pharmaceutical interventions and health policy controls (one lag), lags of mobility (one lag), lagged new cases, (one lag), lagged NO_2_ and CO emissions (one lag) country-specific time trends, as well as country and time fixed effects. Standard errors are clustered at the country level. ***, **, and * represent statistically significant at 1, 5, and 10%, respectively

**Table 11:** Nonlinear effects of vaccines on economic activity using CO

Variables	(1)	(2)	(3)	(4)	(5)	(6)
	CO	CO	CO	CO	CO	CO
First dose per capita	− 0.001680**	− 0.001771**	0.000410			
	(0.001)	(0.001)	(0.001)			
First dose squared	0.000032**	0.000035*				
	(0.000)	(0.000)				
Second dose per capita		− 0.000303	− 0.001211	0.001004		
		(0.001)	(0.002)	(0.002)		
Second dose squared			0.000039*			
			(0.000)			
Surprises in vaccines administered (per capita)				− 0.001468	− 0.007415	
				(0.001)	(0.007)	
Second dose per capita * Surprises in vaccines administered				0.000032		
				(0.000)		
*Interaction with second dose quartiles (1st quartile omitted)*						
2nd Quartile of second doses administered * Surprises in vaccines					0.008883	
					(0.006)	
3rd Quartile of second doses administered * Surprises in vaccines					0.009174	
					(0.007)	
4th Quartile of second doses administered * Surprises in vaccines					0.008483	
					(0.007)	
Low second doses administered * Surprises in vaccines						− 0.002158
						(0.004)
High second doses administered * Surprises in vaccines						0.001272
						(0.001)
Observations	6208	6208	6208	5861	5861	5861
R-squared	0.282	0.282	0.281	0.278	0.280	0.278
Country FE	Yes	Yes	Yes	Yes	Yes	Yes
Time FE	Yes	Yes	Yes	Yes	Yes	Yes
No. of countries	46	46	46	44	44	44
*P*-value F-test						0.513

Table reports results for the nonlinear impact of vaccines on CO._._ Columns 1 through 3 add additional terms for the square of the share of population that is vaccinated to the specification in Eq. (1). Column 4 through 6 test use different specification to test for interactions between the share of population fully vaccinated and the vaccine surprise variable. Column 4 uses a simple interaction term, column 5 is based on different quartiles of the vaccination rate (Eq. 2), while column 6 allows for interactions based on a smooth transition function. The regressions control for stringency of containment measures, other non-pharmaceutical interventions and health policy controls (one lag), lags of mobility (one lag), lagged new cases, (one lag), lagged NO2 and CO emissions (one lag) country-specific time trends, as well as country and time fixed effects. Standard errors are clustered at the country level. ***, **, and * represent statistically significant at 1, 5, and 10%, respectively

**Table 12:** Nonlinear effects of vaccines on economic activity using mobility

Variables	(1)	(2)	(3)	(4)	(5)	(6)
	Mobility	Mobility	Mobility	Mobility	Mobility	Mobility
First dose per capita	− 0.208008	− 0.111641	0.068691			
	(0.138)	(0.164)	(0.090)			
First dose squared	0.005015*	0.002153				
	(0.003)	(0.003)				
Second dose per capita		0.322693**	0.060115	0.128190		
		(0.160)	(0.270)	(0.208)		
Second dose squared			0.006777			
			(0.004)			
Surprises in vaccines administered (per capita)				0.084600	0.498283	
				(0.574)	(0.396)	
Second dose per capita * Surprises in vaccines administered				0.008895		
				(0.008)		
*Interaction with second dose quartiles (1st quartile omitted)*						
2nd Quartile of second doses administered * surprises in vaccines					− 0.298942	
					(0.505)	
3rd quartile of second doses administered * surprises in vaccines					− 0.035946	
					(0.454)	
4th Quartile of second doses administered * surprises in vaccines					0.127307	
					(0.382)	
Low second doses administered * Surprises in vaccines						− 0.204844
						(0.590)
High second doses administered * Surprises in vaccines						0.687222***
						(0.175)
Observations	6215	6215	6215	5868	5868	5868
R-squared	0.435	0.438	0.439	0.444	0.444	0.444
Country FE	Yes	Yes	Yes	Yes	Yes	Yes
Time FE	Yes	Yes	Yes	Yes	Yes	Yes
No. of countries	46	46	46	44	44	44
*P*-value F-test						0.136

Table reports results for the nonlinear impact of vaccines on retail and recreational mobility_2._ Columns 1 through 3 add additional terms for the square of the share of population that is vaccinated to the specification in Eq. (1). Column 4 through 6 test use different specification to test for interactions between the share of population fully vaccinated and the vaccine surprise variable. Column 4 uses a simple interaction term, column 5 is based on different quartiles of the vaccination rate (Eq. 2), while column 6 allows for interactions based on a smooth transition function. The regressions control for stringency of containment measures, other non-pharmaceutical interventions and health policy controls (one lag), lags of mobility (one lag), lagged new cases, (one lag), lagged NO_2_ and CO emissions (one lag) country-specific time trends, as well as country and time fixed effects. Standard errors are clustered at the country level. ***, **, and * represent statistically significant at 1, 5, and 10%, respectively

**Table 13:** Effect of vaccines—role of containment and new cases using CO and Mobility

Variables	(1)	(2)	(3)	(4)
	CO	CO	Retail	Retail
Surprises in vaccines administered (per capita)	0.000330	0.001720	0.717312***	0.995808***
	(0.001)	(0.002)	(0.147)	(0.367)
*Interaction with stringency measures quartiles (1st quartile omitted)*				
2nd quartile of containment measures * surprises in vaccines	0.000709		− 0.191644	
	(0.000)		(0.131)	
3rd quartile of containment measures * surprises in vaccines	0.000208		− 0.431621**	
	(0.001)		(0.179)	
4th quartile of containment measures * surprises in vaccines	− 0.001011		− 0.384407*	
	(0.001)		(0.227)	
*Interaction with new cases quartiles (1st quartile omitted)*				
2nd quartile of new cases * surprises in vaccines		− 0.001192		− 0.402936
		(0.002)		(0.354)
3rd quartile of new cases * surprises in vaccines		− 0.001486		− 0.513714
		(0.002)		(0.382)
4th quartile of new cases * surprises in vaccines		− 0.001395		− 0.440691
		(0.002)		(0.350)
Observations	5861	5861	5839	5839
R-squared	0.278	0.278	0.449	0.452
Country FE	Yes	Yes	Yes	Yes
Time FE	Yes	Yes	Yes	Yes
Country-time trend	Yes	Yes	Yes	Yes
No. of countries	44	44	44	44

Table reports results for Eq. 3. The dependent variable is change in CO per capita in columns 1 and 2 and change in retail and recreational mobility in columns 3 and 4. The forecast error in vaccine rollout is interacted with the stringency of containment measures (categorized into four quartiles) in column 1 and 3. The forecast error in vaccine rollout is interacted with the level of new cases (moving average over seven days and also categorized into four quartiles) in column 2 and 4. The variables are lagged one day. All regressions control for stringency of containment measures and other non-pharmaceutical interventions, lagged mobility and NO_2_ per capita, country specific time trends, as well as country and time fixed effects. Standard errors are clustered at the country level. ****p* < 0.01, ***p* < 0.05, **p* < 0.1

## Abbreviations

NO_2_: Nitrogen dioxide
CO: Carbon monoxide

## Footnotes

1 https://covid.ourworldindata.org/

2 https://github.com/CSSEGISandData/COVID-19

3 https://www.airfinity.com/

4 All regressions control for country and time fixed effects, and standard errors are clustered at the country level. The significance of the results remain unchanged if estimated using Driscoll-Kraay standard errors that allow for more general cross-sectional and temporal dependence.

5 http://waqi.info/

6 https://www.google.com/covid19/mobility/index.html

7 https://covidtracker.bsg.ox.ac.uk/

8 It can be argued that controlling for NPIs may bias the results downwards if NPIs are affected by vaccinations. While we are primarily interested in the partial effect of vaccinations after controlling for NPIs, our results continue to hold if we exclude NPIs as control.

9 Results are qualitatively similar when using simple interaction terms instead of the semi-parametric specification described in Eq. 3.

10 As the pandemic has impacted trade very differently across sectors (e.g. tourism has been adversely impacted while trade in medical goods and electronics has boomed), the spillover effects may also depend on the structure of trade between countries. For example, more cases or lockdowns in a trading partner which imports medical goods or electronics may have less of a negative impact than a trading partner which imports agricultural and mining products. While these effects would be interesting to explore, we leave this for future research.

11 Throughout the paper we use the vaccine surprise variable as an independent variable, similar to the use of fiscal policy shocks in Ramey (2011), Auerbach and Gorodnichenko (2013) and Ramey and Zubairy (2018). However, the results are broadly similar if we use vaccine surprises as an instrument for the percent of population that is fully vaccinated (Appendix Table 9). See Ramey (2016) for a discussion on using macroeconomic variables as shocks or instruments in VAR and Local Projection settings.

12 We further test the residual term for unit root and cointegration tests reject the hypothesis of no cointegration.

13 The quantitative comparison between the impact of vaccines and containment measures on economic activity should be interpreted with caution as this assumes a linear impact of both variables on economic activity. We find that vaccines have nonlinear effects (see below) on economic activity, and potential nonlinearities associated with the impact of containment measures makes such direct comparisons problematic. In addition, containment might be endogenous to vaccinations despite additional controls, making such a comparison difficult.

14 In principle, the economic benefits from vaccines may hit diminishing returns once vaccination rates hit high enough levels, especially as a country approaches herd immunity. We add cubic terms to the regression to test this hypothesis but do not find any evidence for diminishing returns. This result may potentially reflect the fact that not enough countries have reached high enough vaccination rates to approach herd immunity, in part because the new, more transmissible, variants of the virus may have raised herd immunity thresholds.

15 Appendix Table 13 uses CO (columns 1 and 2) and mobility (column 3 and 4) as the dependent variable for similar interaction regressions. The results are generally weaker for these measures of economic activity.

16 US vaccines’ spillover term is calculated by multiplying US vaccines with each country’s bilateral trade exposure to the USA.

17 As the number of countries with high vaccination rates remain limited at the time of writing, the paper was not able to explore the potential for diminishing returns to vaccines at high levels of vaccination. Exploring higher order nonlinear effects (over and above the quadratic term shown in the paper) could be an interesting avenue for future research. If returns to vaccine were to diminish after a certain point, then this would add another rationale for sharing vaccine doses more equitably across countries.

## References

[CR1] Agarwal, R., & Gita G. (2021). A proposal to end the COVID-19 pandemic. *IMF Staff Discussion Notes 2021, no. 004*.

[CR2] Auerbach AJ, Gorodnichenko Y (2013). Output spillovers from fiscal policy. American Economic Review.

[CR3] Carvalho, V. M., Hansen, S., Ortiz, A., Garcia, J. R., Rodrigo, T., Rodriguez Mora, S., & Ruiz de Aguirre, P. (2020). Tracking the COVID-19 crisis with high-resolution transaction data. 10.1098/rsos.210218 PMC8355671 34401194

[CR4] Coibion, O., Gorodnichenko, Y., & Weber, M. (2020). The cost of the covid-19 crisis: Lockdowns, macroeconomic expectations, and consumer spending (No. w27141). National Bureau of Economic Research.

[CR5] Dagan, N., Barda, N., Kepten, E., Miron, O., Perchik, S., Katz, M. A., Hernán, M. A., Lipsitch, M., Reis, B., & Balicer, R. (2021). BNT162b2 mRNA Covid-19 Vaccine in a Nationwide Mass Vaccination Setting. N Engl J Med, NEJMoa2101765. 10.1056/NEJMoa2101765 PMC7944975 33626250

[CR6] Deb, P., Furceri, D., Ostry, J. D., & Nour, T. (2020a). The effects of containment measures on the COVID-19 pandemic. *IMF Working Paper, 20/159.*

[CR7] Deb, P., Furceri, D., Ostry, J. D., & Nour, T. (2020b). The economic effects of COVID-19 containment measures. *IMF Working Paper, 20/158*.

[CR8] Deb, P., Furceri, D., Jimenez, D., Kothari, S., Ostry, J. D., & Nour, T. (2021a). Determinants of COVID-19 Vaccine Rollouts and Their Effects on Health Outcomes. *IMF Working Paper, XX/XXX*. 10.1007/s40258-022-00757-6 PMC9470512 36100820

[CR300] Deb, P., Furceri, D., Jimenez, D., Kothari, S., Ostry, J. & Tawk, N. (2021d). 'Determinants of COVID-19 Vaccine Rollouts and Their Effects on Health Outcomes'. London, Centre for Economic Policy Research. https://cepr.org/active/publications/discussion_papers/dp.php?dpno=16681. 10.1007/s40258-022-00757-6 PMC9470512 36100820

[CR9] Deb, P., Furceri, D., Ostry, J. D., Tawk, N., & Naihan, Y. (2021c). Effects of fiscal measures during COVID-19. *IMF Working Paper, XX/XXX.*

[CR10] Deb, P., Ganslmeier, M., Furceri, D., Ostry, J. D., & Nour, T. (2021). Vaccinate early and vaccinate broadly: On the health and economic effects of COVID-19 Vaccines. https://www.researchsquare.com/article/rs-525515/v1

[CR11] Fernández-Villaverde, J., & Charles I. J. (2020). Macroeconomic outcomes and COVID-19: A progress report. No. w28004. National Bureau of Economic Research.

[CR12] International Monetary Fund. (2020a). The great lockdown: Dissecting the economic effects. World Economic Outlook, Chapter 2. October 2020a.

[CR13] International Monetary Fund. (2020b). COVID-19 lockdowns and exits in asia: some lessons. Asia and Pacific Regional Economic Outlook, Chapter 2. October 2020b.

[CR14] International Monetary Fund. (2021). Leveraging opportunities from COVID-19 vaccines: Early lessons from Asia. Asia and Pacific Regional Economic Outlook, Chapter 3. Washington, DC.

[CR15] Lin J-T, Michael BM (2011). Detection from space of a reduction in anthropogenic emissions of nitrogen oxides during the Chinese economic downturn. Atmospheric Chemistry and Physics.

[CR16] Polack FP, Thomas SJ, Kitchin N, Absalon J, Gurtman A (2020). C4591001 clinical trial group. Safety and efficacy of the BNT162b2 mRNA Covid-19 vaccine. New England Journal of Medicine.

[CR17] Ramey AV (2011). Identifying government spending shocks: It's all in the timing. The Quarterly Journal of Economics.

[CR18] Ramey VA (2016). Macroeconomic shocks and their propagation. Handbook of Macroeconomics.

[CR19] Ramey VA, Zubairy S (2018). Government spending multipliers in good times and in bad: Evidence from US historical data. Journal of Political Economy.

[CR20] Sampi, B., James, R. E., & Charl, J. (2020). Nowcasting economic activity in times of COVID-19: An approximation from the Google Community Mobility Report. World Bank Policy Research Working Paper 9247.

[CR21] Sandmann, F. G., Davies, N. G., Vassall, A., Edmunds, W. J., Jit, M., Sun, F. Y., Villabona-Arenas, C. J. *et al.* (2021). The potential health and economic value of SARS-CoV-2 vaccination alongside physical distancing in the UK: A transmission model-based future scenario analysis and economic evaluation. *The Lancet Infectious Diseases*. 10.1016/S1473-3099(21)00079-7 PMC7972313 33743846

[CR22] Voysey M, Clemens SAC, Madhi SA, Weckx LY, Folegatti PM, Aley PK, Angus B (2021). Safety and efficacy of the ChAdOx1 nCoV-19 vaccine (AZD1222) against SARS-CoV-2: An interim analysis of four randomised controlled trials in Brazil, South Africa, and the UK. The Lancet.

